# It’s Never over until It’s over: How Can Age and Ovarian Reserve Be Mathematically Bound through the Measurement of Serum AMH—A Study of 5069 Romanian Women

**DOI:** 10.1371/journal.pone.0125216

**Published:** 2015-04-24

**Authors:** Bogdan Doroftei, Cristina Mambet, Mihaela Zlei

**Affiliations:** 1 Obstetrics and Gynecology Department, Gr. T. Popa University of Medicine and Pharmacy, Iasi, Romania; 2 Origyn Fertility Center, Iasi, Romania; 3 Synevo Central Laboratory-Medicover Group, Bucuresti, Romania; 4 Laboratory of Molecular Biology, Regional Institute of Oncology, Iasi, Romania; China Agricultural University, CHINA

## Abstract

Wide regional differences in the age-related Anti Mullerian hormone (AMH) regression patterns or age at onset of natural menopause have been reported, possibly reflecting genetic, socioeconomic, environmental, racial or ethnic peculiarities. Moreover, adaptation of AMH levels from different assays using regression functions may lack accuracy and externally defined references for AMH levels may not fully comply with a specific geographical area. The current study aimed to establish an accurate mathematical relationship between AMH serum values and age in a large group of women from Romania, as any consistent difference from previously reported regression models may aid in building specific profiles for the AMH decline with age in this geographical region. Our study pointed out to the quadratic regression as the most fitted pattern of correlation for all the age groups between 24 and 45. To our knowledge the current manuscript is based on the singular study carried out in this geographical region, generating a particular age-related pattern of association between age and serum AMH levels in women, regardless of their subjacent pathologies.

## Introduction

Increasing evidence within the worldwide medical field point towards infertility as a growing epidemic-like issue for which prevention and treatment strategies should be urgently initiated. The most frequent concerns women wish to address when seeking preconception counseling in an Assisted Reproductive Technology (ART) unit are: “what chances do we have?” and “is it too late?”. Though nosuch medical facilities use crystal balls to offer instant solutions, several laboratory tests and clinical parameters may be immediately considered and ranked according to their informative value in terms of chances to conception. One of the most reliable tests, when assessing whether the reproductive window is still open for a certain patient, is the serum detection of Anti-Müllerian Hormone (AMH) [1, 2]. Several groups reported a mathematical relationship between serum AMH values and women’s age [3–8] and others extended the use of these findings in search for accurate prediction for the moment of menopause debut [9]. However, wide geographical differences in the age-related AMH decrease pattern and age at onset of natural menopause have been reported, possibly reflecting genetic, socioeconomic, environmental, racial or ethnic peculiarities [10–12]. On the other hand, the lack of consensual standardization of the measurement methodology may require that each ART unit generate its own specific AMH reference values for dealing with their patients in a more appropriate, personalized manner [13].

The purpose of the current study is to establish an accurate mathematical relationship between AMH serum values and age in a large group of women from Romania. The secondary aim of the study is that any consistent difference from previously reported regression models may aid in building of a specific profile for AMH decline with age in this geographical region and to reach a truthful prediction tool for calculating the extent of the reproductive period in our area. leading to an optimized approach of infertility- and menopause-associated conditions in regional ART facilities.

## Materials and Methods

### Study group

5069 Romanian women aged between 18 and 55 years were included in this observational study. All patients were submitted from fertility and gynecology centers and were processed between April 2009—December 2013, in the Synevo Laboratories, Romania, facilities providing centralized AMH testing for a number of medical centers within Romania. Patients with ovarectomy, ovarian cancer or undetectable serum AMH levels (under 0.1 ng/ mL) were not considered eligible for the study. Serum samples were separated by centrifugation from whole blood specimens collected by venous puncture. AMH serum levels were measured by means of an enzymatically based immunoassay (AMH Gen II ELISA kit, Beckman-Coulter) and a fully automated analyzer (DSX analyzer, DYNEX Technologies). The detection method was validated with intra-assay (within run) and inter-assay (between runs) coefficients of variation (CV) of 3.5–5.3% and 5–8.5%, respectively. AMH values were reported as ng/ mL. The lower limit of detection was 0.1 ng/ mL.

### Ethics statement

All clinical investigation has been conducted according to the principles expressed in the Declaration of Helsinki. Written informed consent was obtained from all subjects. The current study was a retrospective, observational study, therefore, we did not consider necessary to seek approval from an ethical committee. At the time of puncture of blood, testing was undertaken for clinical purposes and all patients consented to analysis of data for publication. The data was supplied anonymously and no identifying patient information was disclosed at any point. None of the authors had any direct contact with patients in this study and patient data was anonymized before the authors had access to it.

### Statistics

Data processing was carried out using IBM SPSS Statistics for Window 20. Individual AMH values were used to determine median, mean, and standard deviation (SD) at one-year intervals. The Pearson test was used to assess the correlation between two variables (age and AMH serum level), with age as the independent variable (predictor) and the AMH value as the dependent variable. The Pearson test was used also to evaluate the data for normality of distribution. Regression analysis was intended to determine whether age-related changes in AMH concentrations were best fitted by a linear or quadratic function. For each age, seven empirical percentiles (3rd, 10th, 25th, 50th, 75th, 90th, and 97th) were applied.

## Results

The mean value of serum AMH levels within the studied cohort was 1.85 ng/ mL, the range, 0.1 ng/ mL- 36,21 ng/ mL. Values of mean, median, and SD had an annual decline with advancing age, as shown in Table 1. Thus, by the age of 30, the mean AMH levels decreased by 0.3 ng/ mL per year.Females between 31 and 35 years had mean AMH levels decrease by 0.2 ng/ mL, and, by the age of 50 years, the mean AMH decrease was 0.1 ng/ mL. The median AMH levels decreased with 0.25 ng/ mL per year for patients between 26 and 30, with 0.2 ng/ mL for those between 31 and 36 years, and with 0.1 ng/ mL beyond the age of 36 years. The SD values followed a similar decrease, with 0.15 ng/ mL by the age of 35, and, afterwards, with 0.1 ng/ mL per year.

**Table 1:** Age-specific mean, median, and standard deviation values of serum AMH levels (ng/ mL) for 5069 women from Romanian Infertility Centers.

Average decrease per year (mean)	Age	Number of patients	Mean	Median	SD
	<24	76	5.05	3.91	5.69
	24	42	4.81	3.75	4.70
	25	43	4.32	3.4	3.26
-0.3	26	66	4.15	3.22	3.77
	27	106	3.73	2.92	3.74
	28	159	3.50	2.3	4.00
	29	200	3.12	2.03	3.42
	30	262	2.82	1.93	3.01
-0.2	31	318	2.69	1.95	2.75
	32	333	2.43	1.70	2.55
	33	345	2.14	1.3	2.48
	34	347	1.82	1.2	2.05
	35	367	1.66	0.91	2.11
-0.1	36	372	1.56	0.95	2.02
	37	328	1.18	0.78	1.26
	38	293	1.13	0.59	1.56
	39	252	1.09	0.57	1.52
	40	235	0.94	0.48	1.16
-0.1	41	200	0.67	0.23	1.04
	42	207	0.85	0.36	1.45
	43	173	0.47	0.1	0.62
	44	161	0.6	0.1	1.98
	45	79	0.23	0.1	0.28
-0.1	46	27	0.28	0.1	0.39
	47	26	0.21	0.1	0.23
	48	22	0.14	0.1	0.16
	49	14	0.12	0.1	0.04
	50	7	0.14	0.1	0.10
-0.1	>50	9	0.13	0.1	0.094

**Fig 1:**
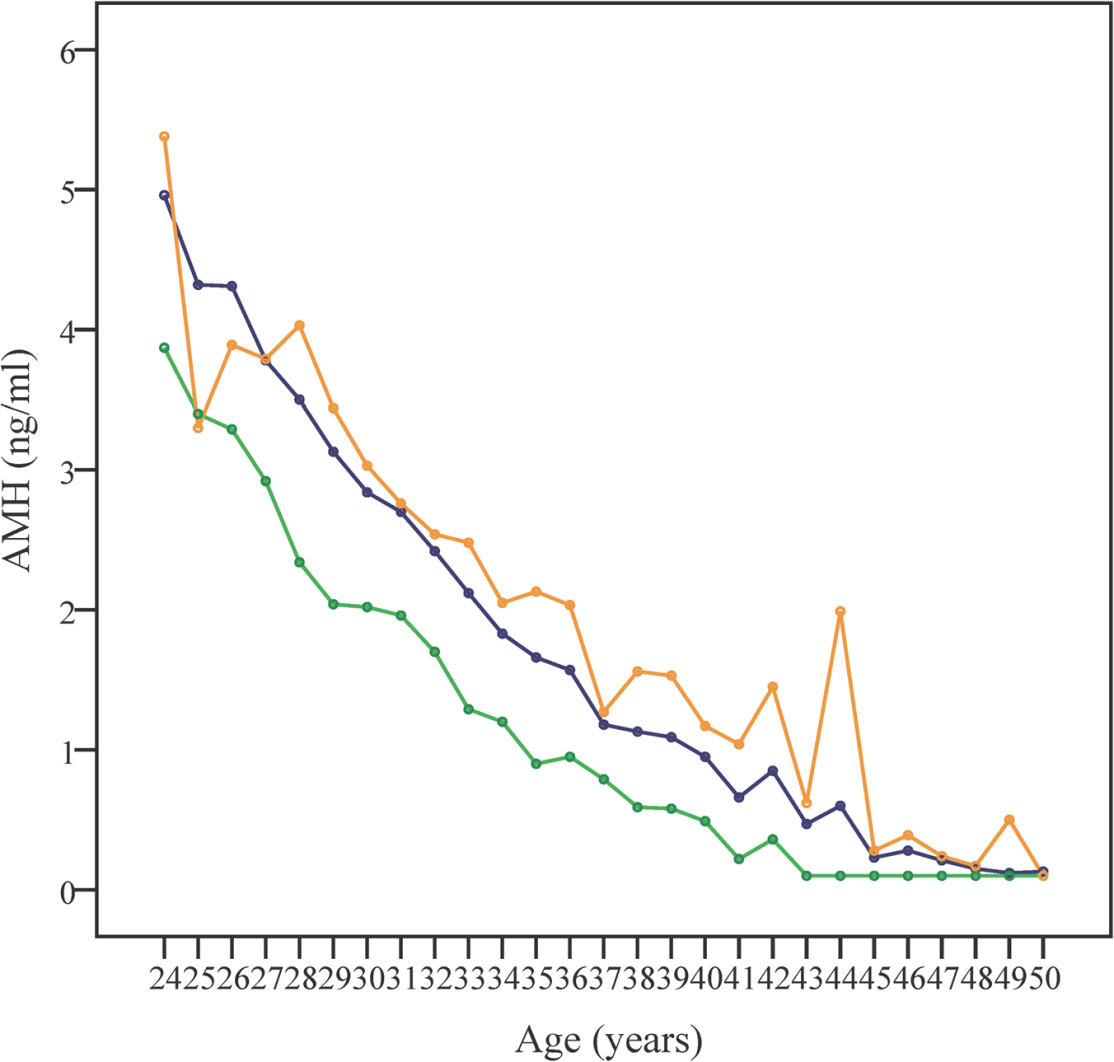
Representation of age-specific AMH median, mean, and SD values at one year age intervals. Serum AMH values decline in direct relationship to aging. Mean (blue), median (green) and standard deviation (SD, orange) of AMH values are represented versus age (years).

**Fig 2:**
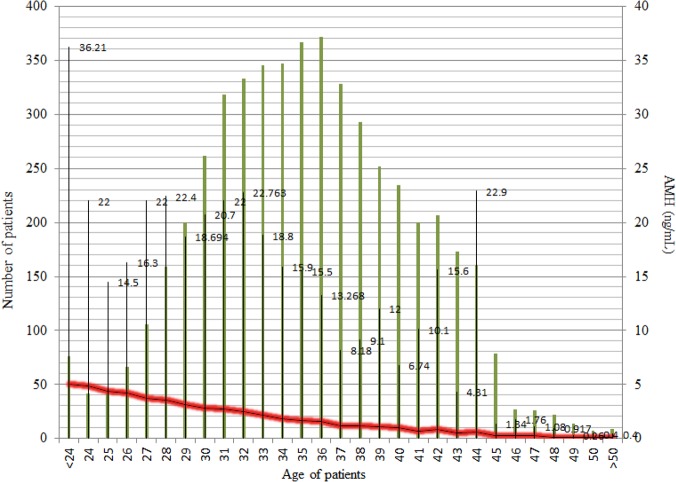
The decline pattern of serum AMH levels in relationship to aging in the studied group. Patients (n = 5069) were grouped based on their age (green lines); the maximum AMH levels were given in numbers; the red line merges the mean value of every group.

**Fig 3:**
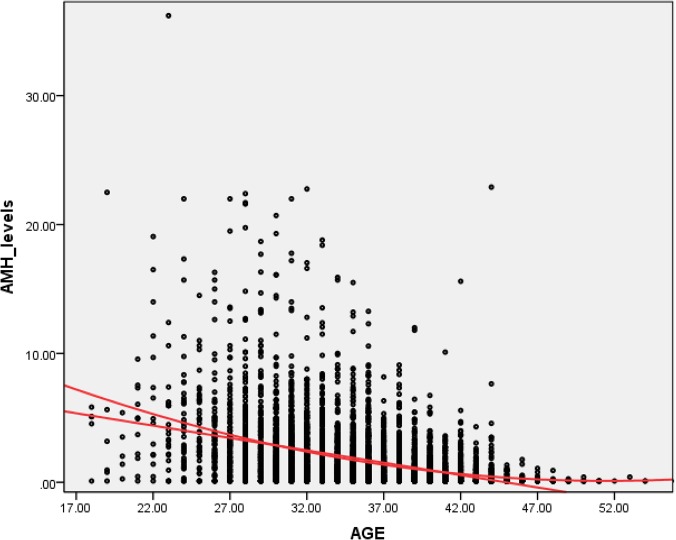
The asymmetrical, right-tailed skewness of AMH level distribution against age (in years).

The decline pattern of serum AMH levels in relationship to aging is depicted in Fig 1, where mean, median, and SD values were represented. Both Figs 1 and 2 show the difference between mean and median, suggesting a skew over the upper side of the graph. The calculated skewness value is 3.60, positive, thus the distribution was asymmetrical, right tailed, as shown in Fig 3.

For the variables age and mean of AMH values, a Pearson coefficient of correlation (r) of—0.963 was obtained, p = 0.01, and N = 29. The variation ratio was R^2^ = r*r = 0.963*0.963*100 = 92.7%, meaning that this particular correlation is true for 92.7% of females from our studied cohort. A significant correlation was calculated between age and AMH medians (r = - 0.934, and R^2^ = 87.23%) and between age and AMH SDs (r = -0.951, R^2^ = 90.44%). The negative r values (- 0.963;- 0.934;- 0.951) showed that low values of age were associated with high values of AMH, medium values of age with medium values of AMH, and high values of age with low values of AMH. The variation ratios for mean, median and SD were 92.73%, 87.23%, and 90.44%, respectively, revealing that more than 87% of the patients present the same declining pattern for serum AMH levels in correlation with age.

**Fig 4:**
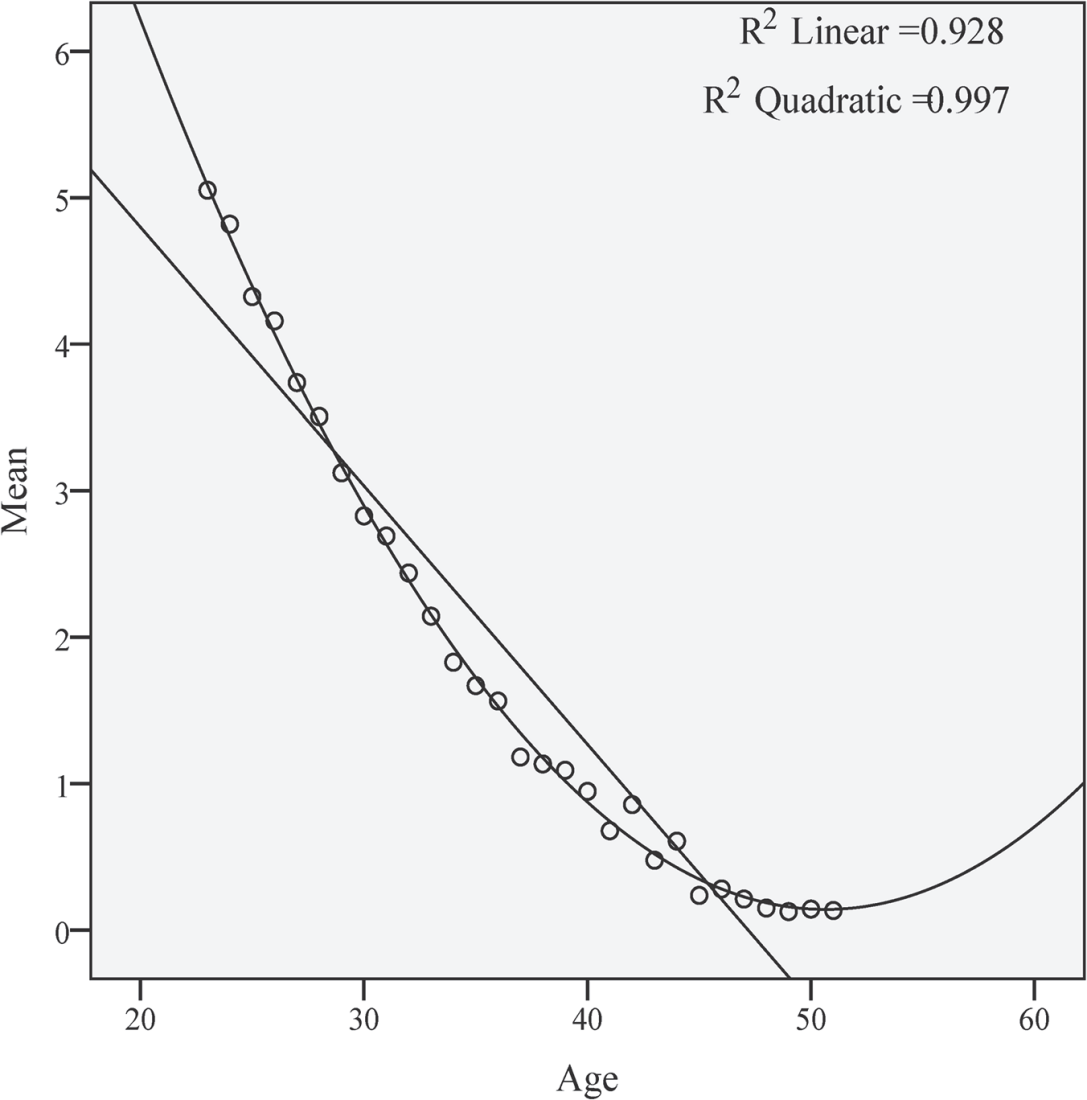
The quadratic regression of the AMH mean values is more useful to illustrate the AMH decline with age (in years).

**Fig 5:**
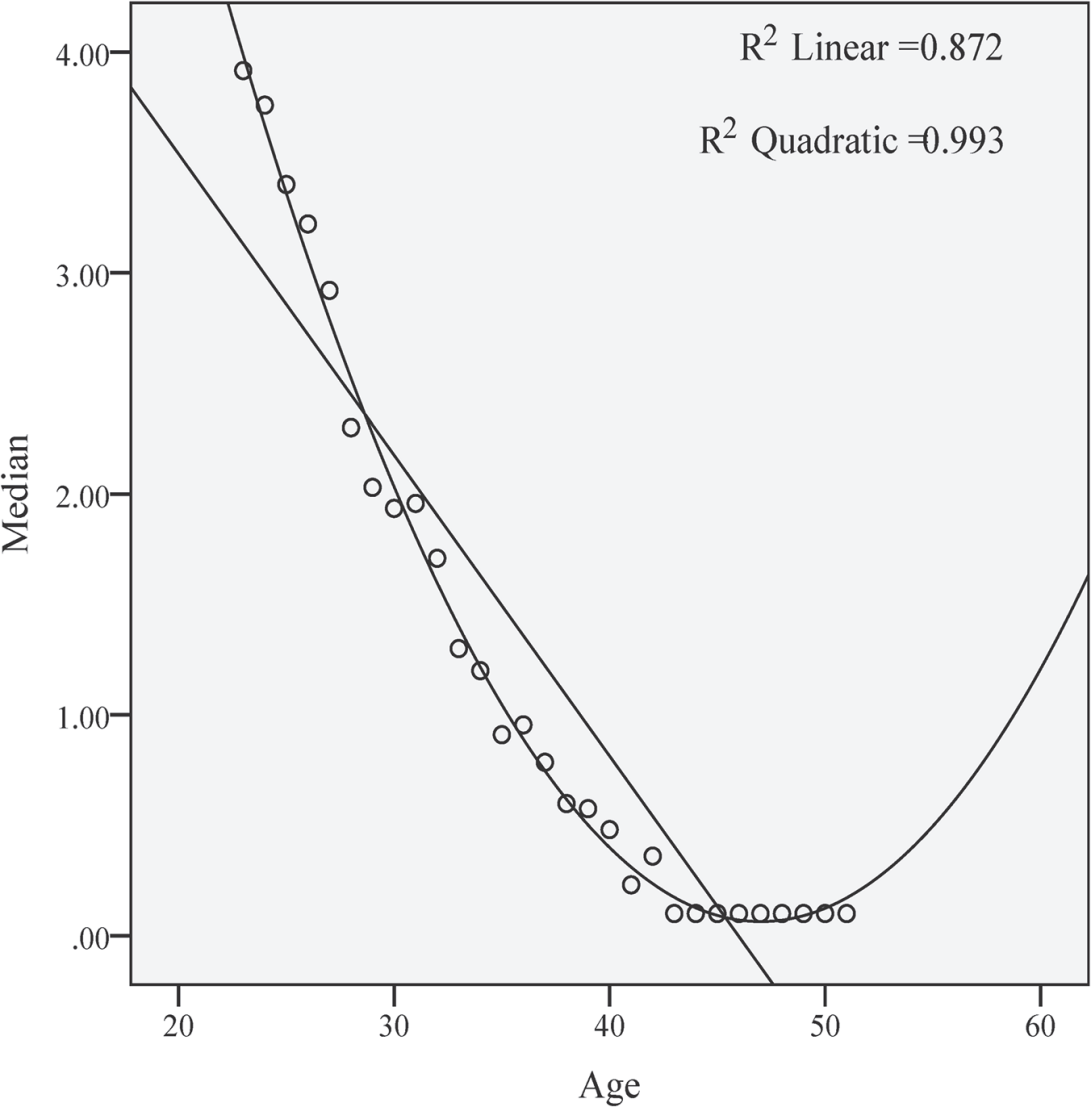
The quadratic regression versus linear regression of the AMH median values.

Using the linear and quadratic equations for correlating the regressions, both mean (Fig 4) and median (Fig 5) AMH values showed a simultaneous decline with increasing age. The quadratic regression fits better to the form of the curve, both for mean (R^2^ quadratic = 0.997 versus R^2^ linear = 0.928) and for the median (R^2^ quadratic = 0.993 versus R^2^ linear = 0.872). The adjusted R^2^, revealing the power of prediction of the model, for mean is R^2^ linear = 0.925. Using ANOVA, the note F was 4452.65, whereas the residual note was 0.198. The residue values of mean, revealing the deviations from the real model, are ranging between—0.61 and 0.80, thus our model predicts the AMH levels with a deviation of ± 0.41.

**Fig 6:**
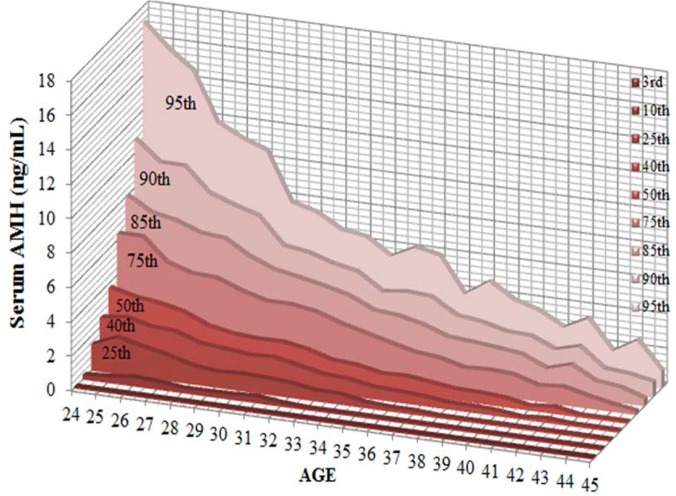
Correlation between the 3rd, 10th, 25th, 50th, 75th, 90th, and 97th percentiles of serum AMH level and age.

Using the data from women between ages of 24 to 45, we built a normogram that includes the 3rd, 10th, 25th, 40th, 50th, 75th, 85th, 90th, and 95th percentiles of serum AMH level correlated with aging. Data are shown in Table 2 and the normogram is depicted in Fig 6.

**Table 2:** Correlation between the 3rd, 10th, 25th, 40th, 50th, 75th, 90th, and 95th percentiles of serum AMH level and age (n = 4888, age between 24 and 45).

		Serum AMH level ng/ mL								
Age	Numberof patients(n = 4888)	3rd	10th	25^th^	40th	50th	75th	85th	90th	95^th^
24	42	0.10	0.26	1.51	2.55	3.76	6.38	8.09	10.84	17.09
25	43	0.10	0.33	2.09	2.73	3.40	6.37	7.25	9.66	15.72
26	66	0.10	0.49	1.74	2.39	3.22	5.06	6.97	9.64	14.65
27	106	0.10	0.41	1.45	2.29	2.92	4.62	6.46	8.27	11.84
28	159	0.10	0.25	1.05	1.81	2.30	4.55	6.32	7.75	11.10
29	200	0.10	0.26	0.88	1.67	2.03	4.01	5.46	7.33	10.60
30	262	0.10	0.29	0.95	1.52	1.94	3.62	4.90	5.81	7.80
31	318	0.10	0.35	0.90	1.58	1.96	3.61	4.65	5.60	7.43
32	333	0.10	0.19	0.80	1.29	1.71	3.39	4.36	5.15	6.61
33	345	0.10	0.10	0.58	0.99	1.30	2.91	4.01	4.88	6.40
34	347	0.10	0.10	0.56	0.90	1.20	2.54	3.35	3.94	5.48
35	367	0.10	0.10	0.30	0.65	0.91	2.18	3.22	4.11	6.19
36	372	0.10	0.10	0.20	0.59	0.96	1.83	2.91	4.00	5.89
37	328	0.10	0.10	0.20	0.51	0.79	1.69	2.40	3.13	3.88
38	293	0.10	0.10	0.10	0.35	0.60	1.33	2.29	2.95	4.80
39	252	0.10	0.10	0.10	0.33	0.58	1.40	2.14	2.59	3.91
40	235	0.10	0.10	0.10	0.27	0.48	1.32	1.98	2.53	3.49
41	200	0.10	0.10	0.10	0.10	0.23	0.88	1.37	1.95	2.73
42	207	0.10	0.10	0.10	0.21	0.36	1.00	1.78	2.32	3.45
43	173	0.10	0.10	0.10	0.10	0.10	0.68	1.01	1.23	1.75
44	161	0.10	0.10	0.10	0.10	0.10	0.40	0.85	1.11	2.58
45	79	0.10	0.10	0.10	0.10	0.10	0.18	0.38	0.84	0.97

## Discussions

The current study relies on data collected from a single laboratory chain and based on an homogeneous assay, involving 5069 Romanian female patients with ages between 18 and 55. The primary aim of this observational study was to define our own normogram, as already pointed out as a necessity by others [13], given the potential peculiarities that may apply for our geographical region. As shown within the results section, the values of serum AMH decreased simultaneously with aging, in a non-linear manner. Higher mean than median values were calculated, suggesting that there are some high values that contribute to a non-normal distribution. This pattern has been previously described [6], in studies involving, like in ours, all patients submitted to fertility and gynecology centers, regardless of their subjacent pathologies, such as the presence of polycysticovary syndrome (PCOS) or infertility/ subfertility. The impact of the presence of PCOS, body mass index (BMI), metabolic indices, adiposity, the ovary volume on serum AMH levels is however still a matter of debate in different geographical areas [14–18].

Our findings show that the age variable is useful in the prediction of the AMH values, although exist some deviations from the reality and there is a tendency to underestimate the low levels and to overestimate the high levels of serum AMH. Nevertheless high quadratic correlation coefficients (0.997; 0.993) sustain the worth of the current model.

Our study points out to the quadratic regression as the most fitted pattern of correlation for all the age groups between 24 and 45, consistent with the findings of Nelson et al [5, 7], whereas others [4] found that for the 3^rd^ to 50^th^ percentiles, AMH correlates with aging in a linear regression and that the curves over the 75^th^ percentile fit best within a cubic equation. In our study, the age-specific mean, median and standard deviation for serum AMH levels suggest a significant decrease in ovarian reserve, especially in women between ages of 25 to 35. The most extensive study published so far (performed on a US group of 17000 women) [6] reported a decline of AMH mean and median values of 0.2 ng/ mL per year for women older than 36, while in our study the decline for this range of age was less pronounced (0,1 ng/ mL). Some differences between our study and those already published may be explained either by genetic, socioeconomic, environmental, racial or ethnic peculiarities [10–12]or by the lack of international standardization for AMH measurement [18], or both. The Beckman Coulter Gen II assay used for all AMH measurements in our study is the most commonly used AMH assay at present, although persistent technical interferences have been noticed [19]. Several studies reported differences in the performance of various commercially available AMH immunoassays, using the Gen II as a reference, re-emphasizing the need for an internationally defined standard [19, 20]. Therefore, adaptation of AMH levels from different assays using regression functions may lack accuracy [20] and externally defined references for AMH levels may not fully comply with a specific geographical area [13].

Additionally, serum AMH level is nowadays being intensively evaluated as a biomarker predicting the outcome (implantation, pregnancy, live birth rates) for in vitro fertilization procedures [13, 21] and it is expected to aid in ART-specific clinical decision making.

## Conclusion

To our knowledge the current manuscript is based on the first and notably extensive study carried out in this geographical region, generating a particular age-related pattern of association between age and serum AMH levels in women, regardless of their subjacent pathologies. Further studies should be performed for analyzing the decline of AMH with age progression in major pathologies, such as PCOS, unilateral ovariectomy, benign or malignant ovarian tumors and its predictive value the outcome of for ART technology.
